# Supplementary material for: A Six-Year Retrospective Study of Microbiological Characteristics and Antimicrobial Resistance in Specimens from a Tertiary Hospital’s Surgical Ward
Source: Antibiotics (Basel). 2023 Mar 1;12(3):490. doi: 10.3390/antibiotics12030490 (PMC10044204; doi:10.3390/antibiotics12030490)
Supplement: Supplementary file 1 [file antibiotics-12-00490-s001.zip › antibiotics-2219626-Supplementary.pdf]

antibiotics

MDPI

Table S1. Microbiological data of the pathogens isolated from patients' samples.

Pathogen	Number of Isolates (% for That Specific Year)						
	**2016**	**2017**	**2018**	**2019**	**2020**	**2021**	**2016-2021**
**Gram-positives**	**84 (51.53)**	**86 (43.65)**	**109 (42.91)**	**112 (48.48)**	**150 (45.05)**	**134 (47.67)**	**675 (46.26)**
Enterococcus faecalis	20 (12.27)	24 (12.18)	27 (10.63)	32 (13.85)	41 (12.31)	19 (6.76)	163 (11.17)
Enterococcus faecium	11 (6.75)	11 (5.58)	12 (4.72)	22 (9.52)	40 (12.01)	33 (11.74)	129 (8.84)
Staphylococcus epidermidis	10 (6.13)	7 (3.55)	19 (7.48)	15 (6.49)	14 (4.20)	22 (7.83)	87 (5.96)
Staphylococcus aureus	6 (3.68)	9 (4.57)	12 (4.72)	3 (1.30)	6 (1.80)	8 (2.85)	44 (3.01)
Streptococcus anginosus	7 (4.29)	7 (3.55)	4 (1.57)	5 (2.16)	4 (1.20)	5 (1.78)	32 (2.19)
Staphylococcus haemolyticus	2 (1.23)	5 (2.54)	3 (1.18)	4 (1.73)	1 (0.30)	8 (2.85)	23 (1.57)
Staphylococcus hominis	3 (1.84)	2 (1.02)	7 (2.76)	3 (1.30)	4 (1.20)	4 (1.42)	23 (1.57)
Streptococcus constellatus	0 (0)	1 (0.51)	4 (1.57)	2 (0.87)	1 (0.30)	8 (2.85)	16 (1.09)
Enterococcus avium	0 (0)	2 (1.02)	2 (0.79)	2 (0.87)	6 (1.80)	2 (0.71)	14 (0.96)
Streptococcus mitis	3 (1.84)	3 (1.52)	2 (0.79)	2 (0.87)	2 (0.60)	1 (0.36)	13 (0.89)
Enterococcus gallinarum	1 (0.61)	3 (1.52)	2 (0.79)	2 (0.87)	4 (1.20)	0 (0)	12 (0.82)
Streptococcus salivarius	3 (1.84)	1 (0.51)	0 (0)	1 (0.43)	3 (0.90)	2 (0.71)	10 (0.69)

Staphylococcus lugdunensis	1 (0.61)	1 (0.51)	2 (0.79)	1 (0.43)	1 (0.30)	2 (0.71)	8 (0.54)
Streptococcus gordonii	0 (0)	0 (0)	2 (0.79)	3 (1.30)	1 (0.30)	1 (0.36)	7 (0.48)
Streptococcus sanguinis	2 (1.23)	1 (0.51)	1 (0.39)	1 (0.43)	1 (0.30)	0 (0)	6 (0.41)
Clostridium perfringens	0 (0)	0 (0)	1 (0.39)	3 (1.30)	1 (0.30)	0 (0)	5 (0.34)
Enterococcus durans	1 (0.61)	0 (0)	1 (0.39)	0 (0)	2 (0.60)	1 (0.36)	5 (0.34)
Staphylococcus coagulase negative	4 (2.45)	1 (0.51)	0 (0)	0 (0)	0 (0)	0 (0)	5 (0.34)
Corynebacterium striatum	0 (0)	0 (0)	0 (0)	0 (0)	3 (0.90)	1 (0.36)	4 (0.27)
Propionibacterium granulosum	1 (0.61)	0 (0)	0 (0)	2 (0.87)	1 (0.30)	0 (0)	4 (0.27)
Corynebacterium amycolatum	0 (0)	0 (0)	0 (0)	0 (0)	1 (0.30)	2 (0.71)	3 (0.21)
Peptostreptococcus anaerobius	1 (0.61)	0 (0)	1 (0.39)	0 (0)	0 (0)	1 (0.36)	3 (0.21)
Streptococcus alactolyticus	0 (0)	1 (0.51)	0 (0)	0 (0)	0 (0)	2 (0.71)	3 (0.21)
Streptococcus gallolyticus	0 (0)	0 (0)	0 (0)	0 (0)	2 (0.60)	1 (0.36)	3 (0.21)
Streptococcus intermedius	1 (0.61)	1 (0.51)	0 (0)	0 (0)	0 (0)	1 (0.36)	3 (0.21)
Streptococcus parasanguinis	0 (0)	0 (0)	1 (0.39)	0 (0)	1 (0.30)	1 (0.36)	3 (0.21)

antibiotics

Actinomyces turicencis	1 (0.61)	0 (0)	0 (0)	0 (0)	0 (0)	1 (0.36)	2 (0.14)
Enterococcus hirae	1 (0.61)	0 (0)	0 (0)	0 (0)	1 (0.30)	0 (0)	2 (0.14)
Finegoldia magna	1 (0.61)	1 (0.51)	0 (0)	0 (0)	0 (0)	0 (0)	2 (0.14)
Micrococcus luteus	0 (0)	0 (0)	0 (0)	0 (0)	2 (0.60)	0 (0)	2 (0.14)
Rothia mucilaginosa	0 (0)	0 (0)	0 (0)	2 (0.87)	0 (0)	0 (0)	2 (0.14)
Staphylococcus cohnii	1 (0.61)	0 (0)	0 (0)	1 (0.43)	0 (0)	0 (0)	2 (0.14)
Staphylococcus saprophyticus	0 (0)	0 (0)	0 (0)	0 (0)	1 (0.30)	1 (0.36)	2 (0.14)
Streptococcus milleri	0 (0)	0 (0)	1 (0.39)	0 (0)	0 (0)	1 (0.36)	2 (0.14)
Actinobaculum schaalii	0 (0)	1 (0.51)	0 (0)	0 (0)	0 (0)	0 (0)	1 (0.07)
Actinomyces odontolyticus	0 (0)	0 (0)	0 (0)	0 (0)	0 (0)	1 (0.36)	1 (0.07)
Anaerococcus vaginalis	0 (0)	0 (0)	0 (0)	0 (0)	0 (0)	1 (0.36)	1 (0.07)
Arcanobacterium haemolyticum	0 (0)	0 (0)	0 (0)	0 (0)	0 (0)	1 (0.36)	1 (0.07)
Bacillus cereus	0 (0)	0 (0)	0 (0)	0 (0)	0 (0)	1 (0.36)	1 (0.07)
Bifidobacterium spp	0 (0)	1 (0.51)	0 (0)	0 (0)	0 (0)	0 (0)	1 (0.07)
Clostridium bifermentans	0 (0)	0 (0)	0 (0)	0 (0)	1 (0.30)	0 (0)	1 (0.07)
Clostridium clostridioforme	0 (0)	0 (0)	0 (0)	0 (0)	0 (0)	1 (0.36)	1 (0.07)
Clostridium difficile	0 (0)	0 (0)	1 (0.39)	0 (0)	0 (0)	0 (0)	1 (0.07)

Clostridium sporogenes	0 (0)	0 (0)	1 (0.39)	0 (0)	0 (0)	0 (0)	1 (0.07)
Corynebacterium jeikeium	0 (0)	0 (0)	0 (0)	0 (0)	1 (0.30)	0 (0)	1 (0.07)
Corynebacterium spp	0 (0)	0 (0)	0 (0)	0 (0)	1 (0.30)	0 (0)	1 (0.07)
Corynebacterium ulcerans	0 (0)	0 (0)	0 (0)	1 (0.43)	0 (0)	0 (0)	1 (0.07)
Eggerthella lenta	0 (0)	0 (0)	1 (0.39)	0 (0)	0 (0)	0 (0)	1 (0.07)
Enterococcus casseliflavus	0 (0)	1 (0.51)	0 (0)	0 (0)	0 (0)	0 (0)	1 (0.07)
Gemella morbillorum	0 (0)	0 (0)	0 (0)	1 (0.43)	0 (0)	0 (0)	1 (0.07)
Lactococcus garvieae	0 (0)	0 (0)	0 (0)	0 (0)	1 (0.30)	0 (0)	1 (0.07)
Parvimonas micra	0 (0)	0 (0)	0 (0)	0 (0)	0 (0)	1 (0.36)	1 (0.07)
Peptoniphilus asaccharolyticus	0 (0)	0 (0)	1 (0.39)	0 (0)	0 (0)	0 (0)	1 (0.07)
Propionibacterium propionicus	1 (0.61)	0 (0)	0 (0)	0 (0)	0 (0)	0 (0)	1 (0.07)
Staphylococcus sciuri	0 (0)	0 (0)	0 (0)	1 (0.43)	0 (0)	0 (0)	1 (0.07)
Staphylococcus warneri	0 (0)	0 (0)	0 (0)	1 (0.43)	0 (0)	0 (0)	1 (0.07)
Streptococcus agalactiae	1 (0.61)	0 (0)	0 (0)	0 (0)	0 (0)	0 (0)	1 (0.07)
Streptococcus dysgalactiae	0 (0)	0 (0)	1 (0.39)	0 (0)	0 (0)	0 (0)	1 (0.07)

Streptococcus gallinarum	0 (0)	0 (0)	0 (0)	0 (0)	1 (0.30)	0 (0)	1 (0.07)
Streptococcus infantarius	0 (0)	0 (0)	0 (0)	0 (0)	1 (0.30)	0 (0)	1 (0.07)
Streptococcus pseudoporcinus	0 (0)	1 (0.51)	0 (0)	0 (0)	0 (0)	0 (0)	1 (0.07)
Streptococcus pyogenes	0 (0)	1 (0.51)	0 (0)	0 (0)	0 (0)	0 (0)	1 (0.07)
Streptococcus viridans	0 (0)	0 (0)	0 (0)	1 (0.43)	0 (0)	0 (0)	1 (0.07)
Turicella otitidis	0 (0)	0 (0)	0 (0)	1 (0.43)	0 (0)	0 (0)	1 (0.07)
Vagococcus fluvialis	1 (0.61)	0 (0)	0 (0)	0 (0)	0 (0)	0 (0)	1 (0.07)
**Gram-negatives**	**71 (43.56)**	**89 (45.18)**	**124 (48.82)**	**99 (42.86)**	**154 (46.25)**	**118 (41.99)**	**655 (44.89)**
Escherichia coli	26 (15.95)	30 (15.22)	48 (18.90)	25 (10.82)	43 (12.91)	51 (18.15)	223 (15.28)
Pseudomonas aeruginosa	6 (3.68)	20 (10.15)	15 (5.91)	11 (4.76)	19 (5.71)	16 (5.69)	87 (5.96)
Enterobacter cloacae	7 (4.29)	11 (5.58)	10 (3.94)	9 (3.90)	17 (5.11)	9 (3.20)	63 (4.31)
Klebsiella pneumoniae	6 (3.68)	2 (1.02)	3 (1.18)	13 (5.63)	25 (7.51)	12 (4.27)	61 (4.18)
Proteus mirabilis	5 (3.07)	7 (3.55)	12 (4.72)	5 (2.16)	5 (1.50)	3 (1.07)	37 (2.53)
Enterobacter aerogenes	2 (1.23)	4 (2.03)	7 (2.76)	4 (1.73)	5 (1.50)	2 (0.71)	24 (1.64)
Bacteroides fragilis	7 (4.29)	1 (0.51)	8 (3.15)	3 (1.30)	1 (0.30)	1 (0.36)	21 (1.44)
Citrobacter freundii	2 (1.23)	3 (1.52)	3 (1.18)	3 (1.30)	7 (2.10)	0 (0)	18 (1.23)
Acinetobacter baumannii	1 (0.61)	1 (0.51)	1 (0.39)	6 (2.60)	3 (0.90)	4 (1.42)	16 (1.10)
Klebsiella oxytoca	2 (1.23)	1 (0.51)	4 (1.57)	3 (1.30)	4 (1.20)	2 (0.71)	16 (1.10)

antibiotics

MDPI

Morganella morganii	0 (0)	1 (0.51)	2 (0.79)	5 (2.16)	4 (1.20)	4 (1.42)	16 (1.10)
Bacteroides thetaiotaomicron	0 (0)	0 (0)	2 (0.79)	0 (0)	5 (1.50)	1 (0.36)	8 (0.55)
Serratia marcescens	0 (0)	0 (0)	0 (0)	1 (0.43)	1 (0.30)	3 (1.07)	5 (0.34)
Aeromonas hydrophila	0 (0)	0 (0)	0 (0)	1 (0.43)	1 (0.30)	2 (0.71)	4 (0.27)
Bacteroides ovatus	0 (0)	0 (0)	1 (0.39)	2 (0.87)	0 (0)	1 (0.36)	4 (0.27)
Citrobacter braakii	1 (0.61)	1 (0.51)	0 (0)	1 (0.43)	1 (0.30)		4 (0.27)
Citrobacter koseri	1 (0.61)	0 (0)	2 (0.79)	0 (0)	0 (0)	1 (0.36)	4 (0.27)
Fusobacterium nucleatum	0 (0)	1 (0.51)	2 (0.79)	0 (0)	0 (0)	1 (0.36)	4 (0.27)
Stenotrophomonas maltophilia	2 (1.23)	0 (0)	0 (0)	1 (0.43)	1 (0.30)	0 (0)	4 (0.27)
Enterobacter cancerogenus	0 (0)	0 (0)	0 (0)	0 (0)	3 (0.90)	0 (0)	3 (0.21)
Bacteroides xylanisolvens	0 (0)	0 (0)	0 (0)	0 (0)	1 (0.30)	1 (0.36)	2 (0.14)
Chromobacterium violaceum	0 (0)	0 (0)	0 (0)	0 (0)	2 (0.60)	0 (0)	2 (0.14)
Haemophilus parainfluenzae	0 (0)	0 (0)	0 (0)	2 (0.87)	0 (0)	0 (0)	2 (0.14)
Prevotella bivia	0 (0)	1 (0.51)	1 (0.39)	0 (0)	0 (0)	0 (0)	2 (0.14)
Proteus vulgaris	0 (0)	1 (0.51)	0 (0)	1 (0.43)	0 (0)	0 (0)	2 (0.14)
Pseudomonas putida	0 (0)	0 (0)	0 (0)	1 (0.43)	1 (0.30)	0 (0)	2 (0.14)
Achromobacter xylosoxidans	0 (0)	0 (0)	1 (0.39)	0 (0)	0 (0)	0 (0)	1 (0.07)

antibiotics

Acinetobacter lwoffi	0 (0)	1 (0.51)	0 (0)	0 (0)	0 (0)	0 (0)	1 (0.07)
Aeromonas punctata (caviae)	0 (0)	0 (0)	0 (0)	0 (0)	0 (0)	1 (0.36)	1 (0.07)
Aeromonas sobria	1 (0.61)	0 (0)	0 (0)	0 (0)	0 (0)	0 (0)	1 (0.07)
Aggregatibacter segnis	0 (0)	0 (0)	0 (0)	1 (0.43)	0 (0)	0 (0)	1 (0.07)
Bacteroides caccae	0 (0)	0 (0)	0 (0)	0 (0)	1 (0.30)	0 (0)	1 (0.07)
Bacteroides eggerthii	0 (0)	0 (0)	0 (0)	0 (0)	1 (0.30)	0 (0)	1 (0.07)
Campylobacter coli	0 (0)	1 (0.51)	0 (0)	0 (0)	0 (0)	0 (0)	1 (0.07)
Citrobacter amalonaticus	0 (0)	1 (0.51)	0 (0)	0 (0)	0 (0)	0 (0)	1 (0.07)
Citrobacter youngae	1 (0.61)	0 (0)	0 (0)	0 (0)	0 (0)		1 (0.07)
Haemophilus segnis	0 (0)	1 (0.51)	0 (0)	0 (0)	0 (0)	0 (0)	1 (0.07)
Klebsiella aerogenes	0 (0)	0 (0)	0 (0)	0 (0)	0 (0)	1 (0.36)	1 (0.07)
Lelliottia amnigena	0 (0)	0 (0)	0 (0)	0 (0)	0 (0)	1 (0.36)	1 (0.07)
Prevotella melaninogenica	0 (0)	0 (0)	0 (0)	0 (0)	1 (0.30)	0 (0)	1 (0.07)
Prevotella oralis	0 (0)	0 (0)	1 (0.39)	0 (0)	0 (0)	0 (0)	1 (0.07)
Proteus hauseri	0 (0)	0 (0)	0 (0)	1 (0.43)	0 (0)	0 (0)	1 (0.07)
Providencia stuartii	1 (0.61)	0 (0)	0 (0)	0 (0)	0 (0)	0 (0)	1 (0.07)
Shewanella algae	0 (0)	0 (0)	0 (0)	0 (0)	1 (0.30)	0 (0)	1 (0.07)
Sphingomonas paucimobilis	0 (0)	0 (0)	0 (0)	0 (0)	1 (0.30)	0 (0)	1 (0.07)
Veillonella parvula	0 (0)	0 (0)	0 (0)	0 (0)	0 (0)	1 (0.36)	1 (0.07)
Veillonella spp	0 (0)	0 (0)	1 (0.39)	0 (0)	0 (0)	0 (0)	1 (0.07)
**Fungi**	**8 (4.91)**	**22 (11.17)**	**21 (8.27)**	**20 (8.66)**	**29 (8.71)**	**29 (10.32)**	**129 (8.84)**

Candida albicans	3 (1.84)	14 (7.11)	10 (3.94)	9 (3.90)	18 (5.41)	18 (6.41)	72 (4.93)
Candida glabrata	2 (1.23)	0 (0)	1 (0.39)	7 (3.03)	8 (2.40)	2 (0.71)	20 (1.37)
Candida tropicalis	3 (1.84)	5 (2.54)	5 (1.97)	1 (0.43)	1 (0.30)	3 (1.07)	18 (1.23)
Candida parapsilosis	0 (0)	2 (1.02)	2 (0.79)	2 (0.87)	1 (0.30)	4 (1.42)	11 (0.75)
Candida guilliermondii	0 (0)	0 (0)	0 (0)	0 (0)	1 (0.30)	1 (0.36)	2 (0.14)
Candida krusei	0 (0)	0 (0)	1 (0.39)	1 (0.43)	0 (0)	0 (0)	2 (0.14)
Candida lusitaniae	0 (0)	0 (0)	1 (0.39)	0 (0)	0 (0)	1 (0.36)	2 (0.14)
Candida ciferrii	0 (0)	0 (0)	1 (0.39)	0 (0)	0 (0)	0 (0)	1 (0.07)
Candida kefyr	0 (0)	1 (0.51)	0 (0)	0 (0)	0 (0)	0 (0)	1 (0.07)
**Number of pathogens (% of total isolates)**	163 (11.2)	197 (13.5)	254 (17.4)	231 (15.8)	333 (22.8)	281 (19.3)	1,459 (100)

Table set S2. Antimicrobial resistance of the most common microorganisms isolated from patients' samples.

Enterococcus faecalis	**Antimicrobial Resistance (%)**
Ampicillin	4,938272
Chloramphenicol	11,538460
Ciprofloxacin	34,868420
Clindamycin	100,000000
Erythromycin	40,789470
Fusidic acid	15,384610
Gentamicin	100,000000
Gentamicin (High Level Synergy)	32,876710
Imipenem	4,098361
Meropenem	0,000000
Moxifloxacin	31,788080
Oxacillin	100,000000
Penicillin	15,950920
Piperacillin	0,000000
Piperacillin/tazobactam	0,000000
Teicoplanin	1,226994
Tetracycline	73,076920
Ticarcillin	100,000000
Ticarcillin+Clavulanic acid	0,000000
Tigecycline	0,000000
Tobramycin	100,000000
Trimethoprim+Sulfamethoxazole	100,000000
Vancomycin	3,680982

Enterococcus faecium	**Antimicrobial resistance (%)**
Ampicillin	79,527560
Cefuroxime	100,000000
Chloramphenicol	0,000000
Ciprofloxacin	83,486240
Clindamycin	100,000000
Erythromycin	83,636360
Fusidic acid	0,000000
Gentamicin	100,000000
Gentamicin (High Level Synergy)	46,153850
Imipenem	84,337350
Levofloxacin	0,000000
Moxifloxacin	81,818180
Oxacillin	100,000000
Penicillin	85,826770
Teicoplanin	32,283470
Tetracycline	34,615380
Tigecycline	3,296703
Tobramycin	100,000000
Trimethoprim+Sulfamethoxazole	100,000000
Vancomycin	33,858270

Staphylococcus epidermidis	**Antimicrobial resistance (%)**
Cefuroxime	100,000000
Chloramphenicol	0,000000
Ciprofloxacin	50,000000
Clindamycin	55,844150
Erythromycin	73,529410
Fusidic acid	85,135130
Gentamicin	24,418600
Imipenem	100,000000
Levofloxacin	53,731340
Oxacillin	84,705880
Penicillin	98,823530
Teicoplanin	0,000000
Tetracycline	18,666670
Tigecycline	0,000000
Tobramycin	22,580640
Trimethoprim+Sulfamethoxazole	27,272730
Vancomycin	0,000000

Staphylococcus aureus	**Antimicrobial resistance (%)**
Amikacin	0,000000
Chloramphenicol	10,000000
Clindamycin	25,641030
Erythromycin	25,641030
Fusidic acid	21,052630
Gentamicin	9,090909
Levofloxacin	37,037040
Moxifloxacin	28,571430
Oxacillin	34,883720
Penicillin	81,395350
Teicoplanin	0,000000
Tetracycline	10,256410
Tigecycline	0,000000
Tobramycin	28,571430
Trimethoprim+Sulfamethoxazole	2,564103
Vancomycin	0,000000

antibiotics MDPI

Streptococcus anginosus	**Antimicrobial resistance (%)**
Ampicillin	0,000000
Cefuroxime	0,000000
Chloramphenicol	0,000000
Ciprofloxacin	3,333333
Clindamycin	23,333330
Erythromycin	23,333330
Fusidic acid	0,000000
Gentamicin	93,333340
Gentamicin (High Level Synergy)	0,000000
Moxifloxacin	0,000000
Oxacillin	100,000000
Penicillin	7,142857
Teicoplanin	0,000000
Tetracycline	43,333330
Tigecycline	0,000000
Tobramycin	100,000000
Trimethoprim+Sulfamethoxazole	13,333330
Vancomycin	0,000000

Staphylococcus haemolyticus	**Antimicrobial resistance (%)**
Chloramphenicol	0,000000
Clindamycin	88,235290
Erythromycin	87,500000
Fusidic acid	76,470590
Gentamicin	60,869560
Levofloxacin	100,000000
Moxifloxacin	22,222220
Oxacillin	95,652180
Penicillin	100,000000
Teicoplanin	0,000000
Tetracycline	5,882353
Tigecycline	0,000000
Tobramycin	55,555560
Trimethoprim+Sulfamethoxazole	58,823530
Vancomycin	0,000000

Staphylococcus hominis	**Antimicrobial resistance (%)**
Chloramphenicol	0,000000
Clindamycin	70,000000
Erythromycin	84,210530
Fusidic acid	84,210530
Gentamicin	8,695652
Levofloxacin	52,631580
Metronidazole	100,000000
Moxifloxacin	20,000000
Oxacillin	81,818180
Penicillin	95,652180
Teicoplanin	0,000000
Tetracycline	20,000000
Tigecycline	0,000000
Tobramycin	60,000000
Trimethoprim+Sulfamethoxazole	35,000000
Vancomycin	0,000000

Streptococcus constellatus	**Antimicrobial resistance (%)**
Ampicillin	0,000000
Cefuroxime	0,000000
Chloramphenicol	0,000000
Ciprofloxacin	6,250000
Clindamycin	12,500000
Erythromycin	18,750000
Fusidic acid	0,000000
Gentamicin	87,500000
Gentamicin (High Level Synergy)	0,000000
Moxifloxacin	0,000000
Oxacillin	100,000000
Penicillin	6,250000
Teicoplanin	0,000000
Tetracycline	18,750000
Tigecycline	0,000000
Tobramycin	100,000000
Trimethoprim+Sulfamethoxazole	0,000000
Vancomycin	0,000000

Enterococcus avium	**Antimicrobial resistance (%)**
Amikacin	100,000000
Ampicillin	0,000000
Chloramphenicol	0,000000
Ciprofloxacin	7,142857
Clindamycin	100,000000
Erythromycin	7,142857
Fusidic acid	37,500000
Gentamicin	100,000000
Gentamicin (High Level Synergy)	0,000000
Imipenem	0,000000
Moxifloxacin	7,142857
Penicillin	14,285710
Teicoplanin	0,000000
Tetracycline	50,000000
Tigecycline	0,000000
Tobramycin	100,000000
Trimethoprim+Sulfamethoxazole	100,000000
Vancomycin	0,000000

Streptococcus mitis	**Antimicrobial resistance (%)**
Ampicillin	9,090909
Cefuroxime	0,000000
Chloramphenicol	0,000000
Ciprofloxacin	50,000000
Clindamycin	16,666670
Erythromycin	16,666670
Fusidic acid	8,333333
Gentamicin	100,000000
Gentamicin (High Level Synergy)	0,000000
Moxifloxacin	0,000000
Oxacillin	100,000000
Penicillin	16,666670
Teicoplanin	0,000000
Tetracycline	16,666670
Tigecycline	0,000000
Tobramycin	100,000000
Trimethoprim+Sulfamethoxazole	8,333333
Vancomycin	0,000000

Enterococcus gallinarum	**Antimicrobial resistance (%)**
Ampicillin	33,333330
Chloramphenicol	0,000000
Ciprofloxacin	50,000000
Clindamycin	85,714290
Erythromycin	33,333330
Fusidic acid	100,000000
Gentamicin	100,000000
Gentamicin (High Level Synergy)	25,000000
Imipenem	45,454540
Moxifloxacin	41,666670
Penicillin	33,333330
Teicoplanin	58,333330
Tetracycline	0,000000
Tigecycline	0,000000
Tobramycin	100,000000
Trimethoprim+Sulfamethoxazole	83,333340
Vancomycin	83,333340

Streptococcus salivarius	**Antimicrobial resistance (%)**
Ampicillin	0,000000
Cefuroxime	0,000000
Chloramphenicol	0,000000
Ciprofloxacin	11,111110
Clindamycin	0,000000
Erythromycin	55,555560
Fusidic acid	0,000000
Gentamicin	77,777780
Gentamicin (High Level Synergy)	0,000000
Moxifloxacin	0,000000
Oxacillin	100,000000
Penicillin	33,333330
Teicoplanin	0,000000
Tetracycline	0,000000
Tigecycline	0,000000
Tobramycin	100,000000
Trimethoprim+Sulfamethoxazole	44,444440
Vancomycin	0,000000

Staphylococcus lugdunensis	**Antimicrobial resistance (%)**
Chloramphenicol	0,000000
Clindamycin	16,666670
Erythromycin	16,666670
Fusidic acid	0,000000
Gentamicin	0,000000
Levofloxacin	0,000000
Moxifloxacin	0,000000
Oxacillin	0,000000
Penicillin	50,000000
Teicoplanin	0,000000
Tetracycline	0,000000
Tigecycline	0,000000
Tobramycin	0,000000
Trimethoprim+Sulfamethoxazole	0,000000
Vancomycin	0,000000

Escherichia coli	**Antimicrobial resistance (%)**
Amikacin	6,726458
Amoxicillin+Clavulanic acid	25,570780
Ampicillin	52,017940
Aztreonam	14,798210
Cefalotin	27,450980
Cefepime	12,612610
Cefotaxime	14,864870
Cefoxitin	11,616160
Ceftazidime	14,349780
Ceftriaxone	15,246640
Cefuroxime	17,488790
Chloramphenicol	8,968610
Ciprofloxacin	29,797980
Colistin	0,000000
Gentamicin	10,454550
Imipenem	1,010101
Levofloxacin	29,608940
Meropenem	0,9049774
Metronidazole	50,000000
Moxifloxacin	29,292930
Penicillin	100,000000
Piperacillin	47,712420
Piperacillin/tazobactam	8,163265

Tetracycline	45,454540
Ticarcillin	50,657890
Ticarcillin+Clavulanic acid	16,339870
Tigecycline	0,5076142
Tobramycin	14,349780
Trimethoprim+Sulfamethoxazole	28,282830

Pseudomonas aeruginosa	**Antimicrobial resistance (%)**
Amikacin	2,298851
Amoxicillin+Clavulanic acid	20,000000
Ampicillin	33,333330
Aztreonam	21,428570
Cefalotin	66,666660
Cefepime	13,793100
Cefotaxime	95,652180
Cefoxitin	100,000000
Ceftazidime	14,942530
Ceftriaxone	91,304340
Cefuroxime	33,333330
Chloramphenicol	25,000000
Ciprofloxacin	22,500000
Clindamycin	50,000000
Colistin	1,162791
Erythromycin	50,000000
Fusidic acid	0,000000
Gentamicin	1,149425
Gentamicin (High Level Synergy)	0,000000
Imipenem	12,500000
Levofloxacin	100,000000
Meropenem	10,344830
Moxifloxacin	39,130440

Penicillin	50,000000
Piperacillin	15,116280
Piperacillin/tazobactam	8,450705
Tetracycline	25,000000
Ticarcillin	31,034480
Ticarcillin+Clavulanic acid	27,848100
Tigecycline	100,000000
Tobramycin	2,325581
Trimethoprim+Sulfamethoxazole	98,734180

Enterobacter cloacae	**Antimicrobial resistance (%)**
Amikacin	4,761905
Amoxicillin+Clavulanic acid	100,000000
Ampicillin	100,000000
Aztreonam	39,682540
Cefalotin	100,000000
Cefepime	14,285710
Cefotaxime	41,935480
Cefoxitin	100,000000
Ceftazidime	39,682540
Ceftriaxone	44,444440
Cefuroxime	96,825390
Chloramphenicol	4,761905
Ciprofloxacin	0,000000
Colistin	1,612903
Gentamicin	0,000000
Imipenem	6,896552
Levofloxacin	0,000000
Meropenem	6,349206
Moxifloxacin	1,724138
Piperacillin	43,181820
Piperacillin/tazobactam	39,655170
Tetracycline	12,068970
Ticarcillin	50,000000

Ticarcillin+Clavulanic acid	48,837210
Tigecycline	5,263158
Tobramycin	4,761905
Trimethoprim+Sulfamethoxazole	3,448276

Klebsiella pneumoniae	**Antimicrobial resistance (%)**
Amikacin	28,947370
Amoxicillin+Clavulanic acid	40,259740
Ampicillin	100,000000
Aztreonam	34,615380
Cefalotin	50,909090
Cefepime	34,615380
Cefotaxime	33,766240
Cefoxitin	33,333330
Ceftazidime	34,615380
Ceftriaxone	34,615380
Cefuroxime	39,743590
Chloramphenicol	34,615380
Ciprofloxacin	33,333330
Colistin	10,389610
Gentamicin	10,256410
Imipenem	28,985510
Levofloxacin	36,206900
Meropenem	30,769230
Moxifloxacin	33,333330
Piperacillin	94,545460
Piperacillin/tazobactam	37,681160
Tetracycline	17,391300
Ticarcillin	100,000000

Ticarcillin+Clavulanic acid	43,396220
Tigecycline	11,940300
Tobramycin	32,051280
Trimethoprim+Sulfamethoxazole	21,739130

Proteus mirabilis	**Antimicrobial resistance (%)**
Amikacin	0,000000
Amoxicillin+Clavulanic acid	14,285710
Ampicillin	37,837840
Aztreonam	8,108109
Cefalotin	6,451613
Cefepime	8,108109
Cefotaxime	8,108109
Cefoxitin	5,882353
Ceftazidime	8,108109
Ceftriaxone	8,108109
Cefuroxime	10,810810
Chloramphenicol	18,918920
Ciprofloxacin	14,705880
Colistin	100,000000
Gentamicin	11,111110
Imipenem	2,941176
Levofloxacin	6,451613
Meropenem	2,702703
Moxifloxacin	44,117650
Piperacillin	22,580640
Piperacillin/tazobactam	3,030303
Tetracycline	100,000000
Ticarcillin	35,483870

Ticarcillin+Clavulanic acid	3,225806
Tigecycline	94,117650
Tobramycin	5,405406
Trimethoprim+Sulfamethoxazole	29,411760

Enterobacter aerogenes	**Antimicrobial resistance (%)**
Amikacin	0,000000
Amoxicillin+Clavulanic acid	100,000000
Ampicillin	100,000000
Aztreonam	29,166670
Cefalotin	100,000000
Cefepime	8,333333
Cefotaxime	29,166670
Cefoxitin	100,000000
Ceftazidime	37,500000
Ceftriaxone	37,500000
Cefuroxime	100,000000
Chloramphenicol	0,000000
Ciprofloxacin	0,000000
Colistin	0,000000
Gentamicin	0,000000
Imipenem	0,000000
Levofloxacin	0,000000
Meropenem	0,000000
Moxifloxacin	0,000000
Piperacillin	26,315790
Piperacillin/tazobactam	29,166670
Tetracycline	0,000000
Ticarcillin	36,842110

Ticarcillin+Clavulanic acid	31,578950
Tigecycline	0,000000
Tobramycin	0,000000
Trimethoprim+Sulfamethoxazole	0,000000

Bacteroides fragilis	**Antimicrobial resistance (%)**
Amoxicillin+Clavulanic acid	5,000000
Ampicillin	100,000000
Cefepime	100,000000
Cefotaxime	95,000000
Cefoxitin	15,000000
Chloramphenicol	0,000000
Clindamycin	50,000000
Erythromycin	80,000000
Metronidazole	21,052630
Penicillin	94,736840
Tetracycline	45,000000
Ticarcillin	75,000000
Ticarcillin+Clavulanic acid	5,000000
Vancomycin	95,000000

Citrobacter freundii	**Antimicrobial resistance (%)**
Amikacin	5,555555
Amoxicillin+Clavulanic acid	100,000000
Ampicillin	100,000000
Aztreonam	27,777780
Cefalotin	100,000000
Cefepime	16,666670
Cefotaxime	27,777780
Cefoxitin	100,000000
Ceftazidime	27,777780
Ceftriaxone	27,777780
Cefuroxime	94,444440
Chloramphenicol	11,111110
Ciprofloxacin	11,111110
Colistin	0,000000
Gentamicin	11,111110
Imipenem	11,111110
Levofloxacin	5,882353
Meropenem	5,882353
Moxifloxacin	11,111110
Piperacillin	30,769230
Piperacillin/tazobactam	17,647060
Tetracycline	0,000000
Ticarcillin	30,769230

Ticarcillin+Clavulanic acid	30,769230
Tigecycline	0,000000
Tobramycin	11,111110
Trimethoprim+Sulfamethoxazole	0,000000

Acinetobacter baumannii	**Antimicrobial resistance (%)**
Amikacin	73,333340
Amoxicillin+Clavulanic acid	100,000000
Ampicillin	100,000000
Aztreonam	93,750000
Cefalotin	100,000000
Cefepime	68,750000
Cefotaxime	68,750000
Cefoxitin	100,000000
Ceftazidime	68,750000
Ceftriaxone	68,750000
Cefuroxime	100,000000
Chloramphenicol	100,000000
Ciprofloxacin	61,538460
Colistin	18,750000
Gentamicin	53,333330
Imipenem	61,538460
Levofloxacin	40,000000
Meropenem	68,750000
Moxifloxacin	61,538460
Piperacillin	90,909090
Piperacillin/tazobactam	66,666660
Tetracycline	61,538460
Ticarcillin	81,818180

Ticarcillin+Clavulanic acid	72,727270
Tigecycline	15,384610
Tobramycin	56,250000
Trimethoprim+Sulfamethoxazole	61,538460

Klebsiella oxytoca	**Antimicrobial resistance (%)**
Amikacin	0,000000
Amoxicillin+Clavulanic acid	0,000000
Ampicillin	100,000000
Aztreonam	6,250000
Cefalotin	8,333333
Cefepime	6,250000
Cefotaxime	6,250000
Cefoxitin	0,000000
Ceftazidime	6,250000
Ceftriaxone	6,250000
Cefuroxime	12,500000
Chloramphenicol	0,000000
Ciprofloxacin	0,000000
Colistin	0,000000
Gentamicin	0,000000
Imipenem	0,000000
Levofloxacin	0,000000
Meropenem	0,000000
Moxifloxacin	0,000000
Piperacillin	100,000000
Piperacillin/tazobactam	0,000000
Tetracycline	0,000000
Ticarcillin	100,000000

Ticarcillin+Clavulanic acid	0,000000
Tigecycline	0,000000
Tobramycin	0,000000
Trimethoprim+Sulfamethoxazole	0,000000

Morganella morganii	**Antimicrobial resistance (%)**
Amikacin	0,000000
Amoxicillin+Clavulanic acid	100,000000
Ampicillin	100,000000
Aztreonam	31,250000
Cefalotin	100,000000
Cefepime	13,333330
Cefotaxime	25,000000
Cefoxitin	33,333330
Ceftazidime	26,666670
Ceftriaxone	25,000000
Cefuroxime	100,000000
Chloramphenicol	18,750000
Ciprofloxacin	26,666670
Colistin	100,000000
Gentamicin	6,250000
Imipenem	0,000000
Levofloxacin	21,428570
Meropenem	0,000000
Moxifloxacin	26,666670
Piperacillin	30,769230
Piperacillin/tazobactam	0,000000
Tetracycline	93,333340
Ticarcillin	30,769230

Ticarcillin+Clavulanic acid	15,384610
Tigecycline	100,000000
Tobramycin	0,000000
Trimethoprim+Sulfamethoxazole	33,333330

antibiotics

MDPI

Bacteroides thetaiotaomicron	**Antimicrobial resistance (%)**
Amoxicillin+Clavulanic acid	50,000000
Ampicillin	87,500000
Cefepime	100,000000
Cefotaxime	87,500000
Cefoxitin	75,000000
Chloramphenicol	0,000000
Clindamycin	66,666660
Erythromycin	75,000000
Metronidazole	12,500000
Penicillin	100,000000
Tetracycline	75,000000
Ticarcillin	87,500000
Ticarcillin+Clavulanic acid	37,500000
Vancomycin	100,000000

Candida albicans	**Antimicrobial resistance (%)**
Amphotericin B	0,000000
Caspofungin	2,777778
Fluconazole	5,555555
Flucytocine	1,388889
Metronidazole	100,000000
Micafungin	2,777778
Voriconazole	0,000000

Candida glabrata	**Antimicrobial resistance (%)**
Amphotericin B	0,000000
Caspofungin	0,000000
Fluconazole	27,777780
Flucytocine	0,000000
Micafungin	0,000000
Voriconazole	5,000000

Candida tropicalis	**Antimicrobial resistance (%)**
Amphotericin B	0,000000
Caspofungin	0,000000
Fluconazole	0,000000
Flucytocine	0,000000
Micafungin	0,000000
Voriconazole	0,000000

Candida parapsilosis	**Antimicrobial resistance (%)**
Amphotericin B	0,000000
Caspofungin	0,000000
Fluconazole	0,000000
Flucytocine	0,000000
Micafungin	0,000000
Voriconazole	0,000000
